# Distribution and prevalence of ixodid tick species (Acari: Ixodidae) infesting cattle in Karamoja region of northeastern Uganda

**DOI:** 10.1186/s12917-023-03802-1

**Published:** 2024-02-07

**Authors:** Patrick Etiang, Abubakar Musoba, David Nalumenya, Christian Ndekezi, Johnson Bbira, Sylvester Ochwo, Robert Tweyongyere, Dennis Muhanguzi

**Affiliations:** 1 College of Veterinary Medicine, Animal Resources and Biosecurity (COVAB), Makerere University, P.O. Box 7062, Kampala, Uganda; 2 Medical Research Council, Uganda Virus Research Institute & London School of Hygiene and Tropical Medicine (MRC/UVRI & LSHTM), Research Unit, P.O. Box 49, Entebbe, Uganda; 3 Center for Animal Health and Food Safety, University of Minnesota, St. Paul, MN 55108 USA

**Keywords:** Karamoja region, Uganda, Ticks, Cattle, morpho-taxonomic keys, *16S* rRNA, *R. microplus*, Tick-borne Diseases

## Abstract

**Background:**

Ticks and tick-borne diseases (TTBDs) are a significant threat to livestock production in sub-Saharan Africa. Transhumance pastoralism practiced in Karamoja region and other factors like cattle trade, communal grazing and the presence of wildlife predispose cattle to TTBDs. Tick species abundance and distribution data can be used as a tool for early disease diagnosis and inform tick control strategies. However, these data for north-eastern Uganda are currently limited; previous surveys were relatively localized and targeted fewer cattle kraals and numbers.

**Methods:**

We randomly collected tick specimens from 1,534 cattle spread across Karamoja region in both the peak month of the rainy (May 2020) and the dry (February2021) seasons. The ticks were identified using morpho-taxonomic keys and the tick species identities confirmed by the *16 S* rRNA gene sequencing and phylogenetic analysis.

**Results:**

A collection of 18,637 ticks was examined and fifteen tick species from three genera (ten *Rhipicephalus;* three *Amblyomma;* two *Hyalomma* species) were identified. *Rhipicephalus appendiculatus* was the most dominant (37.9%) tick species, followed by *Amblyomma variegatum* (32.3%); *A. lepidum* (17.3%); *R. evertsi evertsi* (7.8%); and *R. decoloratus* (1.4%). Eight of these tick species were ubiquitous in the study districts while six were found in isolated areas. The peak month of the dry season collection was associated with a higher proportion of tick-infested cattle (91%) compared to the peak month of the rainy season (89.8%); a difference that was not found statistically significant (χ^*2*^ = 0.5077, n = 1385, p = 0.476). The overall cattle infestation rate was mainly dominated by five tick species namely: *A. variegatum* (55%), *R. appendiculatus* (53%), *A. lepidum* (41%), *R. evertsi* (22%), and *R. decoloratus* (8%). The proportion of tick-infested cattle was highest in Napak District (95.4%) and lowest in Amudat District (80.9%) during the peak month of the rainy season. Napak and Amudat Districts also had the highest and lowest proportion of tick-infested cattle (94.8% and 80.7% respectively) during the peak month of the dry season. *Rhipicephalus microplus* was confirmed in Amudat, Kaabong and Napak districts.

**Conclusion:**

This study demonstrates high tick infestation rates in cattle by a battery of tick species in Karamoja region. We identified both *R. microplus* and *R. decoloratus* which indicates that *R. microplus* has recently been introduced in this region. This calls for effective tick control responses to prevent further spread of this invasive cattle tick specie.

**Supplementary Information:**

The online version contains supplementary material available at 10.1186/s12917-023-03802-1.

## Background

Ticks are the most important vectors of livestock pathogens globally [1, 2]. Additionally, ticks are recognized as the second most important vectors of human pathogens (especially viruses and *Rickettsia*) after mosquitoes [1–3]. The direct effects of ticks on their hosts include metabolic disturbances, toxicosis, stress and immune suppression, whose impact, is seen in the reduction of production, loss of weight, and livestock fatalities [1, 4, 5]. Isolated small surveys of tick species infesting various animal species have been conducted in Uganda over the past 35 years [6]. Most of these surveys did not cover wide areas (for example district-wide) and some used non-random study designs [7–10]. However, the common finding from these surveys was that TTBDs are among the leading constraints of animal health and production in Uganda; particularly in transhumant pastoral regions of Uganda including Karamoja [7]. Surveys carried in Karamoja [7, 8] also reported a high intensity of tick infestation on cattle and wide diversity of tick species. The major tick species infesting cattle in Karamoja region belong to the genera *Rhipicephalus*, *Amblyomma* and *Hyalomma* [7, 8]. Tick-borne diseases (TBDs), especially East Coast fever (ECF) and anaplasmosis, have been reported by Karimojong pastoralists to be the most important because they are associated with high morbidity, mortality and treatment costs [11].

However, most of the surveys conducted in Karamoja used non-random sampling designs and only focused on a few cattle kraals. Such surveys are unlikely to represent area-wide (e.g., district-wide) distributions of tick infestations in cattle. Additionally, the past surveys did not sample cattle in similar localities over the peak months of the rainy and dry season to determine the likely variation of tick abundance | distribution during the two months of May and February in Karamoja region. The distribution and abundance rates of ticks varies across geographical regions, seasons, and it is mainly associated with fluctuations in climatic conditions [10, 12]. Tick species occurrence and distribution in a zone is not only affected by the abiotic factors (vegetation and weather) but also by a host of other factors including the presence of, and movements of the hosts [13]. Given the arid nature of Karamoja, movement of livestock and their herders across the region to exploit the seasonal variation of pastures and water is a key resilience strategy against the harsh climate [14, 15]. Communal grazing and the dry season movement of livestock facilitates tick dispersal and exposes animals to TTBDs [15, 16]. Therefore, it is important to conduct regular surveys to study the occurrence and prevalence of ticks. Given the similarity in morphology of most tick species and the potential damage of ticks during the collection exercise, molecular techniques [17] were used to achieve more precise taxonomic classification of ticks in this study.

Livestock rearing is the key livelihood activity [18] and cattle are the leading livestock type in Karamoja [19]. The cattle population was estimated to be 20% of national total cattle population of 11,408,750 [19]. In addition to their economic importance, cattle are significant in the social and cultural life of the Karimojong pastoralists [14, 18]. The breed of cattle in the region is the short-horned East African Zebu (*Bos indicus*). They are more resistant to tick infestation than *Bos taurus* cattle [20, 21] and are better at regulating their body temperatures, hence requiring less body water. This makes them adapted to the arid conditions of Karamoja that are characterized by prolonged dry spells and high temperatures [20]. Despite the reported high rates of TTBDs in the region [7, 22, 23], the problem remains under-studied. Systematic surveys to generate tick data; which is requisite in the design of control programs, is significant. In this study, we combined morpho-taxonomic keys and molecular tools to provide a more accurate information of tick species infesting cattle over a large expanse [about of the13, 500 square km of land] of Karamoja region during the peak months of the rainy and dry seasons. Our data enriches the map of tick species infesting cattle in Karamoja region; that is requisite in designing and implementing risk-based TTBDs control programs in the region.

## Methods

### Study area

**Fig. 1:**
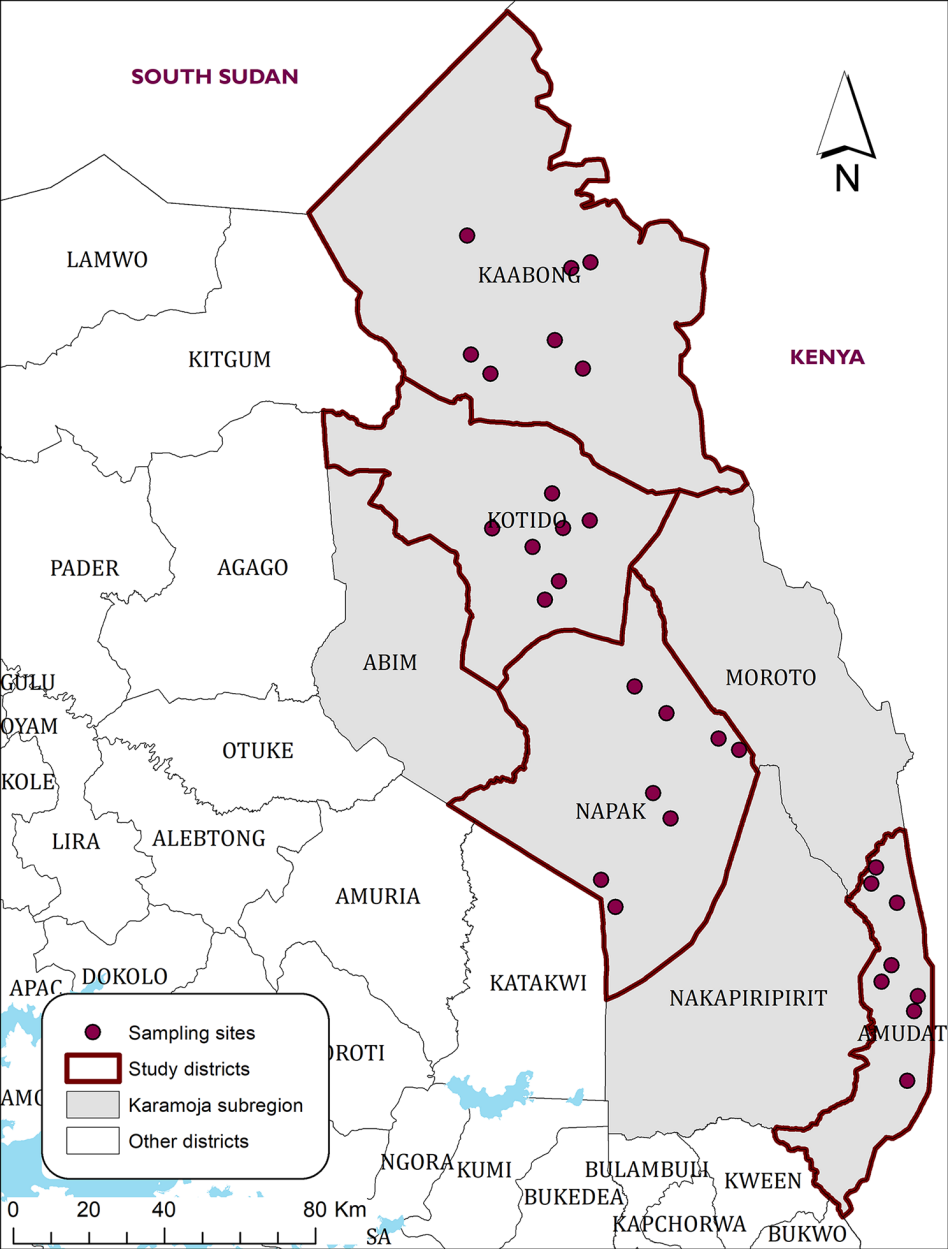
Map of Karamoja region showing the study districts and the sampling sites

This study was conducted in four districts (Kaabong, Kotido, Napak, and Amudat) of Karamoja region in Uganda (Fig. 1). Karamoja region, which is inhabited by the Karimojong, is semi-arid. It has one long rainy period between the months of April to October, and a dry season from November to March. The wettest month is May, and the driest month with high sun intensity is February. The annual average rainfall ranges from 300 mm in the drier northern districts to 1200 mm in western areas of Abim and Nakapiripirit districts. The average annual temperatures range from 16 °C in the highlands to 24 °C in the lowlands. Cattle rearing is the key source of livelihood and pastoralism is the main livestock management system. There is extensive transboundary movement of livestock within districts and/or between countries, mainly Uganda, Kenya, and South Sudan. The districts of Kaabong and Amudat are at a higher altitude characterized by rocky/mountainous landscape. They have a drier climate with minimal crop farming and more cattle rearing. Kotido and Napak lie in the lower plains that drain Kaabong and Moroto respectively. They receive more rainfall and practice some crop farming and cattle rearing. These districts have some of the highest cattle populations in the region. Additionally, they border gazetted wildlife conservation areas, a recipe for TTBD eco-epidemiology, hence warranting regular tick surveys.

### Sampling and individual animal selection

Karamoja region is divided into five administrative divisions (districts, counties/municipalities, sub counties/town councils, parishes/wards, and villages/cells). A stratified multi-stage selection strategy was used to identify the targeted villages for this study. Sampling frames (list of villages, parishes, and sub counties) were obtained from respective district planning units. Using simple random sampling, four districts (out of eight) were selected. In each district, four sub counties were selected and for each sub county, two parishes were selected. One village was selected per parish, and a single kraal sampled per village. In each sampling site, cattle were gathered in central cattle holding grounds or crushes. The cattle were then restrained while standing with aid of ropes and systematic sampling used to pick cows from which ticks were collected (half body tick counts).

### Tick collection and identification

Ticks were collected from 770 cattle during cross-sectional surveys conducted both in the peak month of the rainy (May, 2020) and the dry (February, 2021) seasons in 32 sites. At each site, an average of 20 to 24 cattle were examined. Only ticks visible to the naked eye were collected. Ticks were handpicked from one side of the animal’s body. Ticks from each animal were placed in separate labeled glass vials and preserved with 70% ethyl alcohol. The vials were then transported in a cool box to the Central Diagnostic Laboratory (CDL), Makerere University, Uganda within 7 days of collection. At CDL, ticks were identified to species level under a light stereomicroscope (Olympus™ SZ2-ST Stereomicroscope, Olympus Corporation, Tokyo, Japan) using their morphological characteristics as previously described [24]. Representative ticks of each identified species were selected for genetic validation of the morphological identification.

### DNA extraction

Each tick was cleaned through five one-minute steps of centrifugation at 10,000 rpm in freshly prepared 1.5 ml of phosphate-buffered saline (PBS). Individual clean ticks wrapped in gold foil paper were made brittle by immersion in liquid nitrogen for five minutes and thereafter crushed with a sterile mortar and pestle to generate a tick homogenate. DNA was then extracted from each tick using Qiagen (Qiagen, Hilden, Germany) blood and tissue kits according to the manufacturer’s instructions.

### DNA amplification

PCR amplification of the 455 bp fragment of the 16 S rDNA gene was performed using a single pair of primers 16 S-F: TTA AAT TGC TGT RGT ATT and 16 S-R1: CCG GTC TGA ACT CASAWC and thermo cycling conditions as previously described [17]. Each reaction was prepared in a final volume of 25 µl containing; − 10 µl of 1× PCR buffer, 20 µM of each dNTPs, 1.5 mM MgCl2, 0.25U of Taq Polymerase, 1 µl of 0.2 µM of each primer, 7 µl of nuclease free water, and 5 µl (containing 1ng – 1ug) of genomic DNA template. The amplification was completed in a thermocycler (MultiGene OptiMax Thermal Cycler, Labnet International, Edison, NJ, USA) with initial denaturation of 94° C for 5 min, followed by 35 cycles at 94° C for 30 s, 48° C for 45 s, 72° C for 45 s, and a final extension at 72° C for 7 min. Five microliters of each PCR amplicon stained with ethidium bromide were run on 2% agarose gels and visualised on an ultraviolet transilluminator to check the quality and yield of PCR product. The resultant PCR products were sized against a 1 kb DNA molecular ladder (Bioline, London, UK). PCR products were purified using QIAquick PCR Purification Kit (Qiagen, Hilden, Germany) and commercially Sanger-sequenced (Inqaba Biotec, Pretoria, South Africa).

### Gene sequence analysis

Sequences were manually edited and processed using CLC Main Workbench software v.7.8.1 (CLC bio, Aarhus, Denmark. To reveal the identity of the sequences, each was queried in a BLASTn search tool (NCBI BLASTn software version 2.13.0). The identity of the sequence was assigned to the best hit of the tick species sequences returned with highest identity score (over 90%) and most significant e-value (less than 0.0). The identified query sequences were submitted to the GenBank. Thereafter, sequences obtained from this study, and those downloaded from the GenBank database, were compiled and aligned using MUSCLE [25]. Phylogenetic analysis was performed using maximum likelihood method with 1000 bootstrap replication after best model selection in MEGA v7.0.14 [26]. To evaluate the evolutionary divergence of the queried sequences and those from GenBank, pairwise p-distance comparison and calculation were completed using MEGA 10 software [26] left at default settings for each sequence.

### Data analysis

All statistical data analyses were performed in STATA v15 software (College Station, Texas 77,845 USA). The tick prevalence was determined as number of individuals of a host species infected with a particular tick species divided by number of hosts examined, while the tick mean intensity was determined as the total number of individuals of a particular parasite species in a sample of a host species divided by number of infested individuals of the host species in the sample. The tick relative density or abundance was determined as the total number of individuals of a particular parasite species in a sample of a host species divided by total number of individuals of the host species (infected + uninfected) in the sample. These measures of parasitism were calculated according to the following equations.

1. Prevalence of infestation = number of animals infested with ticks ÷ number of all examined animals × 100.
2. Mean intensity of ticks = total number of collected tick species ÷ number of infested animals in the sample.
3. Relative density of ticks = total number of tick species collected ÷ total number of all animals (infested + uninfested) in the sample.

Differences in tick quantitative indices (tick prevalence and mean intensity) between study districts, and the season of collection were tested using the Chi-square test at a p-value < 0.05 was statistically significant. All prevalence values are reported with their 95% confidence intervals.

## Results

A total of 775 and 756 cattle were examined during the peak months of the rainy (May 2020) and dry (February 2021) seasons, respectively. Of these, only 78 and 68 cattle were found without ticks visible to the naked eye during the respective two seasons. This provided for a proportion of tick-infested cattle of 89.8% (CI: 87.5–91.9%; n = 697) and 91% (CI: 88.7–92.9%; n = 688) in the respective two peak months of the seasons. A total of 7,689 and 10,948 ticks were collected in May 2020 (rainy season) and February 2021 (dry season) respectively. Although the overall proportion of tick-infested cattle in February (dry season) was higher than that of May (rainy season), this difference was not statistically significant (χ^*2*^ = 0.5077, n = 1385, p = 0.476). Similarly, proportions of tick-infested cattle reported per district did not differ significantly between the two seasons (see Additional file 1). The proportion of tick-infested cattle in the rainy season was highest in Napak District at 95.4% (CI: 91.5–97.9%; n = 189) and Kotido District at 92.3% (CI: 87.7–95.6%; n = 182) and lowest in Amudat District at 80.9% (CI: 74.5–86.3%; n = 149), while in the dry season, the proportion of tick-infested cattle was highest in Napak District at 94.8% (CI: 90.8–97.5%; n = 186) and lowest in Amudat District at 80.7% (CI: 73.5–86.7%; n = 122). The tick species, their prevalence rates, and distribution per collection for each district are described in Table 1.

**Table 1:** Distribution of tick species per district and season of collection

Tick genus	Tick species	No. of ticks collected (% of total collection)		Amudat District		Kaabong District		Kotido District		Napak District	
		Rainy season	Dry season	Rainy season	Dry season	Rainy season	Dry season	Rainy season	Dry season	Rainy season	Dry season
*Rhipicephalus*	*R. appendiculatus*	2422	4244	709	771	179	65	550	1189	984	2219
		(32.4)	(41.8)								
	*R. evertsi*	1163	222	71	60	449	18	273	47	370	97
		(15.6)	(2.1)								
	*R. decoloratus*	193	55	9	5	36	1	15	12	133	37
		(2.5)	(0.5)								
	*R. simus*	106	9	6	1	53	0	34	1	13	7
		(1.4)	(0.1)								
	*R. microplus*	37	10	0	2	2	0	0	0	35	8
		(0.5)	(0.1)								
	*R. pravus*	33	28	1	2	8	0	22	20	2	6
		(0.4)	(0.3)								
	*R. pulchellus*	27	15	25	13	0	0	2	1	0	1
		(0.4)	(0.1)								
	*R. geigyi*	17	2	0	0	4	0	8	1	5	1
		(0.2)	(0.01)								
	*R. muhsamae*	12	1	0	1	4	0	8	0	0	0
		(0.2)	(0.01)								
	*R. praetextatus*	1	0	1	0	0	0	0	0	0	0
		(0.01)	(0)								
*Amblyomma*	*A. lepidum*	1997	1054	428	155	27	0	759	628	783	271
		(26.8)	(10.4)								
	*A. variegatum*	1264	4423	148	632	774	2558	107	409	235	824
		(17)	(43.6)								
	*A. gemma*	68	44	68	44	0	0	0	0	0	0
		(0.9)	(0.4)								
*Hyalomma*	*H. truncatum*	91	15	18	8	48	2	15	4	10	1
		(1.2)	(0.1)								
	*H. rufipes*	1	8	0	7	0	0	1	0	0	1
		(0.01)	(0.1)								
Proportion of tick-infested cattle		89.8	91	80.9	80.7	90.3	92.8	92.3	92.9	95.4	94.8
Totals		7432	10,130	1484	1701	1584	2644	1794	2312	2570	3473

**Fig. 2:**
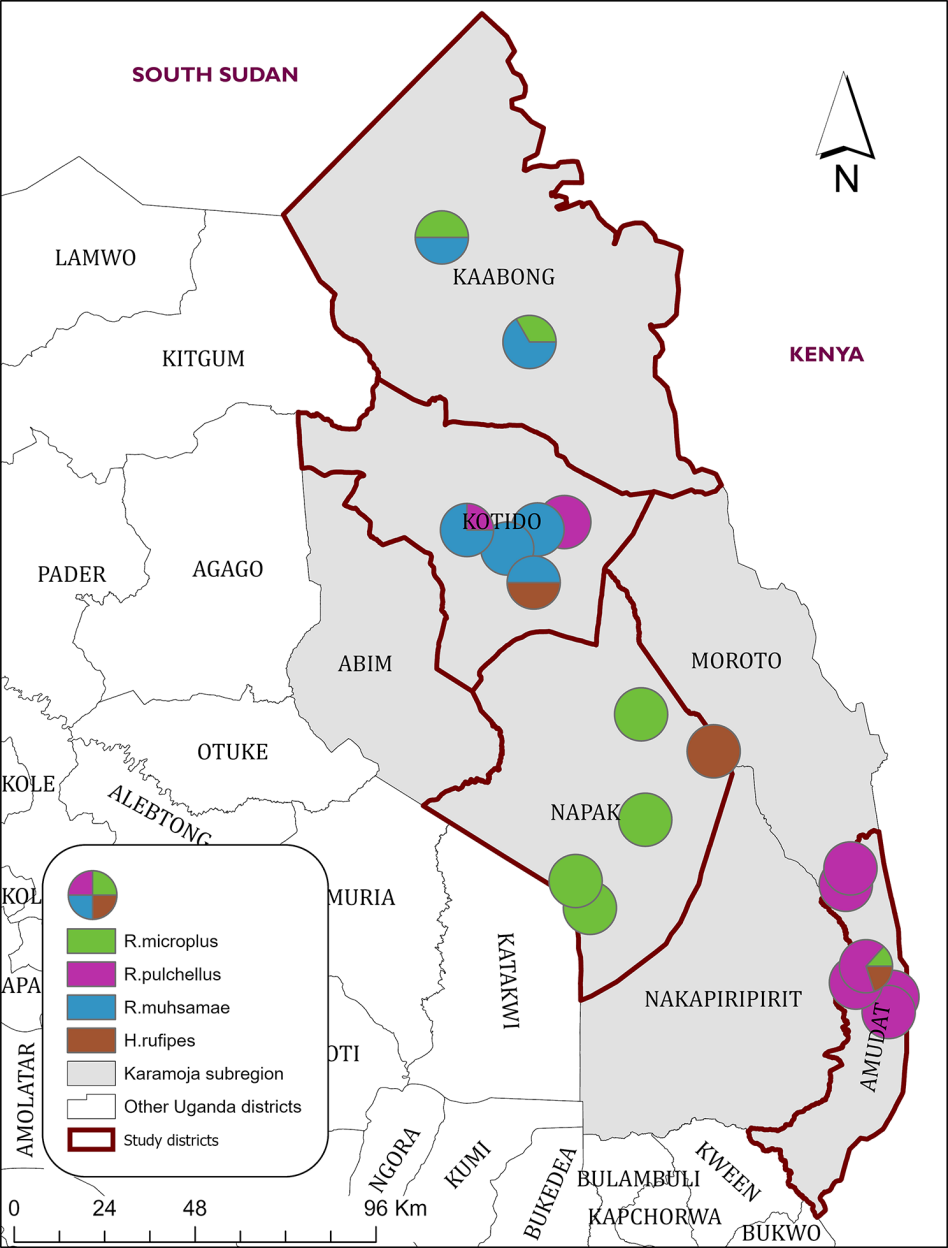
Sampling points that recorded *R. microplus*, *R. pulchellus*, *R. muhsamae* and *H. rufipes*, and their relative dominance

Based on their morphology, a total of fifteen tick species classified into three tick genera were identified in the two season collections. The genera were; *Rhipicephalus* (n = 8,643; 49%), *Amblyomma* (n = 8,891; 50%) and *Hyalomma* (n = 115, 0.65). The most dominant species collected in both rainy and dry seasons collections in descending order were *R. appendiculatus* (6, 666; 37.9%), *A. variegatum* (5,687; 32.3%), A. *lepidum* (3051; 17.3%), *R.evertsi* (1,385; 7.8%), and *R. decoloratus* (248; 1.4%). Additional files show the images of the dominant tick species (see Additional file 2, Additional file 3, Additional file 4, Additional file 5, Additional file 6, Additional file 7, and Additional file 8). The remaining tick species all had dominance rates below 1%. The least dominant tick species were *R. muhsamae* (13; 0.07%), *H. rufipes* (9; 0.05%) and *R. praetextatus* (1; 0.005%):(Table 1). Eight tick species were found in all the four study districts with significant variations in their respective levels of dominance while seven tick species were not detected in all the study districts; *R. microplus* was not detected in Kotido District, *R. pulchellus* was not detected in Kaabong District, *R. muhsamae* and *R. geigyi* were not detected in Amudat District, *H. rufipes* was not detected in Kaabong District while *A. gemma* and *R. praetextatus* were only reported in Amudat District (Fig. 2).

**Fig. 3:**
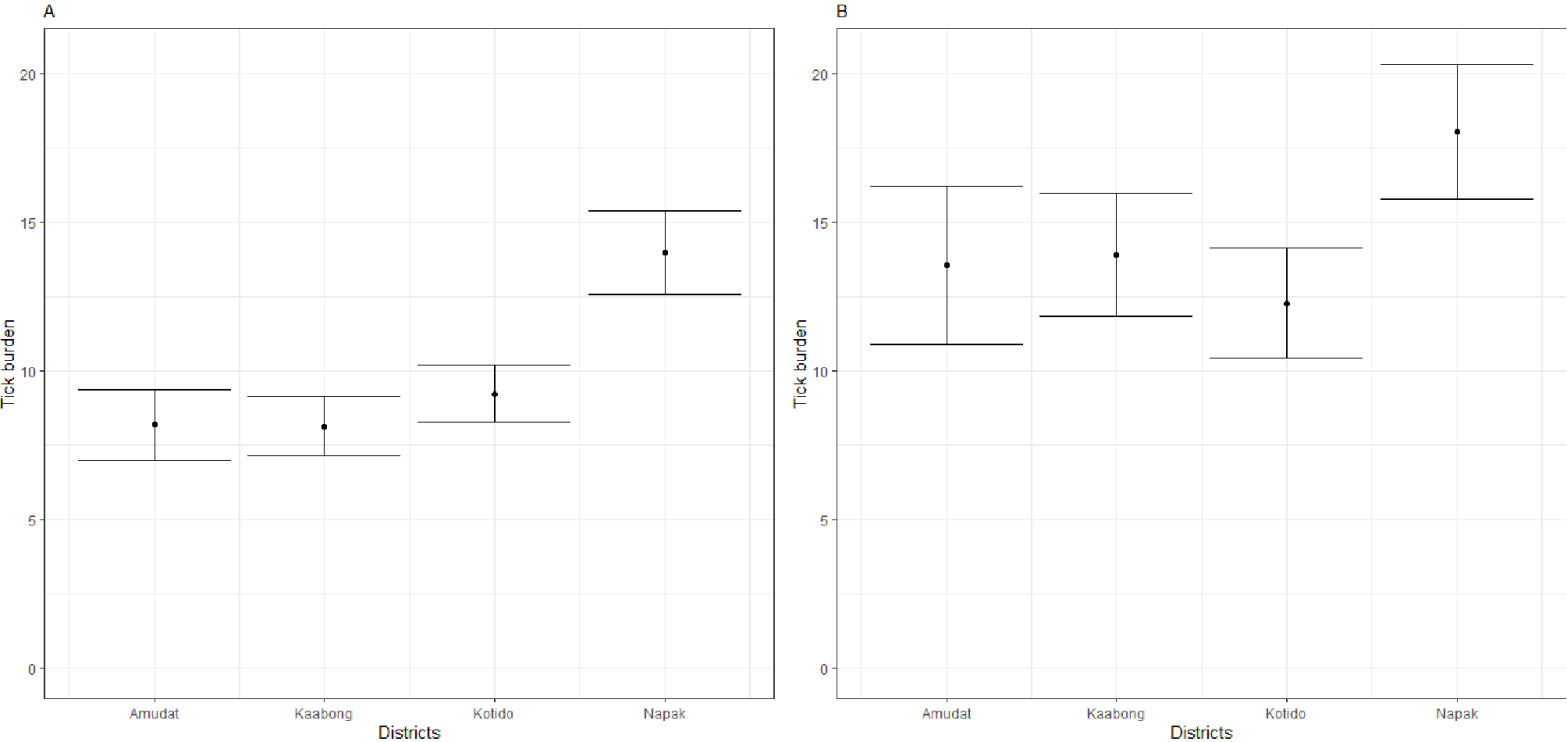
Tick burden on cattle in rainy season (Panel A) and dry season (Panel B). The mean of each district is shown by the black round dot. The bars are the confidence intervals at 95%

The overall average tick counts per cow (all ticks collected) ranged from (8.1, 95% CI: 7.1–9.1) to (13.9, 95% CI: 12.6–15.4); and (12.3, 95% CI: 10.4–14.1) to (18, 95% CI: 15.8–20.3) across all districts in the rainy and dry seasons respectively. Mean tick intensity per animal was significantly higher in Napak District (13.9, 95% CI: 12.6–15.4) compared to the other three study districts that ranged from (8.1, 95% CI: 7.1–9.1) to (9.2, 95% CI: 8.3–10.2) in the rainy season (Fig. 3). Similarly, Napak Districts’ Mean tick intensity was significantly higher (18, 95% CI: 15.8–20.3) compared to the other three study districts that ranged from (12.3, 95% CI: 10.4–14.1) to (13.9, 95% CI: 11.8–15.9) in the dry season (Fig. 3). The tick species prevalence rates, their mean intensity and relative abundance per season of collection are described in Table 2.

**Table 2:** Tick species prevalence rates, mean intensity and relative abundance per peak month of the season of collection

Tick species	Number of cattle infested with tick species		Prevalence (%)		Mean intensity		Abundance	
	Rainy season	Dry season	Rainy season	Dry season	Rainy season	Dry season	Rainy season	Dry season
*R. appendiculatus*	440	378	56.7	49.8	5.6	11.2	3.2	5.6
*R. evertsi*	258	81	33.3	10.7	4.5	2.7	1.5	0.3
*R. decoloratus*	90	35	11.6	4.6	2.1	1.6	0.2	0.1
*R. simus*	62	10	8	1.3	1.7	0.9	0.1	0.01
*R. microplus*	12	6	1.5	0.8	3.0	1.6	0.05	0.01
*R. pravus*	25	24	3.2	3.2	1.3	1.2	0.04	0.04
*R. pulchellus*	20	15	2.6	2.0	1.3	1.0	0.03	0.02
*R. geiygyi*	11	5	1.4	0.6	1.5	0.4	0.02	0.0026
*R. muhsamae*	8	4	1.0	0.5	1.5	0.2	0.02	0.0013
*R. praetextatus*	1	0	0.1	0	1.0	0	0.01	0
* A. lepidum*	370	266	47.7	35.0	5.4	4.0	2.6	1.4
* A. variegatum*	302	557	38.9	73.4	4.2	7.9	1.7	5.8
* A. gemma*	29	26	3.7	3.4	2.3	1.7	0.1	0.06
* H. truncatum*	51	15	6.6	2.0	1.8	1.0	0.1	0.02
* H. rufipes*	1	9	0.1	1.2	1.0	0.9	0.0012	0.01

A significant variation was noted in the ratio of the average number of ticks per cow in the peak month of the rainy to that of the dry season for seven ticks that are major vectors of cattle diseases (Table 3). The average tick counts per cow infested with; *R. evertsi*, *R. decoloratus*, *R. microplus*, *A. lepidum* and *H. rufipes* were higher in the peak month of the rainy season compared to the peak month of the dry season. Conversely the tick counts of *R. appendiculatus* and *A. variegatum* were higher in the peak dry season month compared to the peak month of the rain season (Table 3).

**Table 3:** Ratio of the average tick counts per cow infested with a particular species in the rainy season to the dry season collections

Tick genus	Tick species	Peak Rainy season tick collection	Peak Dry season tick collection	Average tick counts per cow infested with a particular species in Rainy season.	Average tick counts per cow infested with a particular species in Dry season.	Ratio of average tick counts per cow in the two seasons	Comment
*Rhipicephalus*	*R. appendiculatus*	2422	4244	5.5	11.22	1.8	Average tick counts per cow infested with *R. appendiculatus* are 2.03 times more in the dry season compared to the rainy season.
	*R. evertsi*	1163	222	4.5	2.74	5.2	Average tick counts per cow infested with *R. evertsi* are 1.64 times more in the rainy season compared to the dry season.
	*R. decoloratus*	193	55	2.14	1.57	3.5	Average tick counts per cow infested with *R. decoloratus* are 1.36 times more in the rainy season compared to the dry season.
	*R. microplus*	37	10	3.08	1.66	3.7	Average tick counts per cow infested with *R. microplus* are 1.85 times higher in the rainy season compared to the dry season.
*Amblyomma*	*A. lepidum*	1997	1054	5.39	3.96	1.9	Average tick counts per cow infested with *A. lepidum* are 1.36 times higher in the rainy season compared to the dry season.
	*A. variegatum*	1264	4423	4.18	7.94	3.5	Average tick counts per cow infested with *A. variegatum* are 1.89 times higher in the dry season compared to the dry season.
*Hyalomma*	*H. rufipes*	1	8	1	0.88	8	Average tick counts per cow infested with *H. rufipes* are 1.13 times higher in the rain season compared to the dry season.

**Fig. 4:**
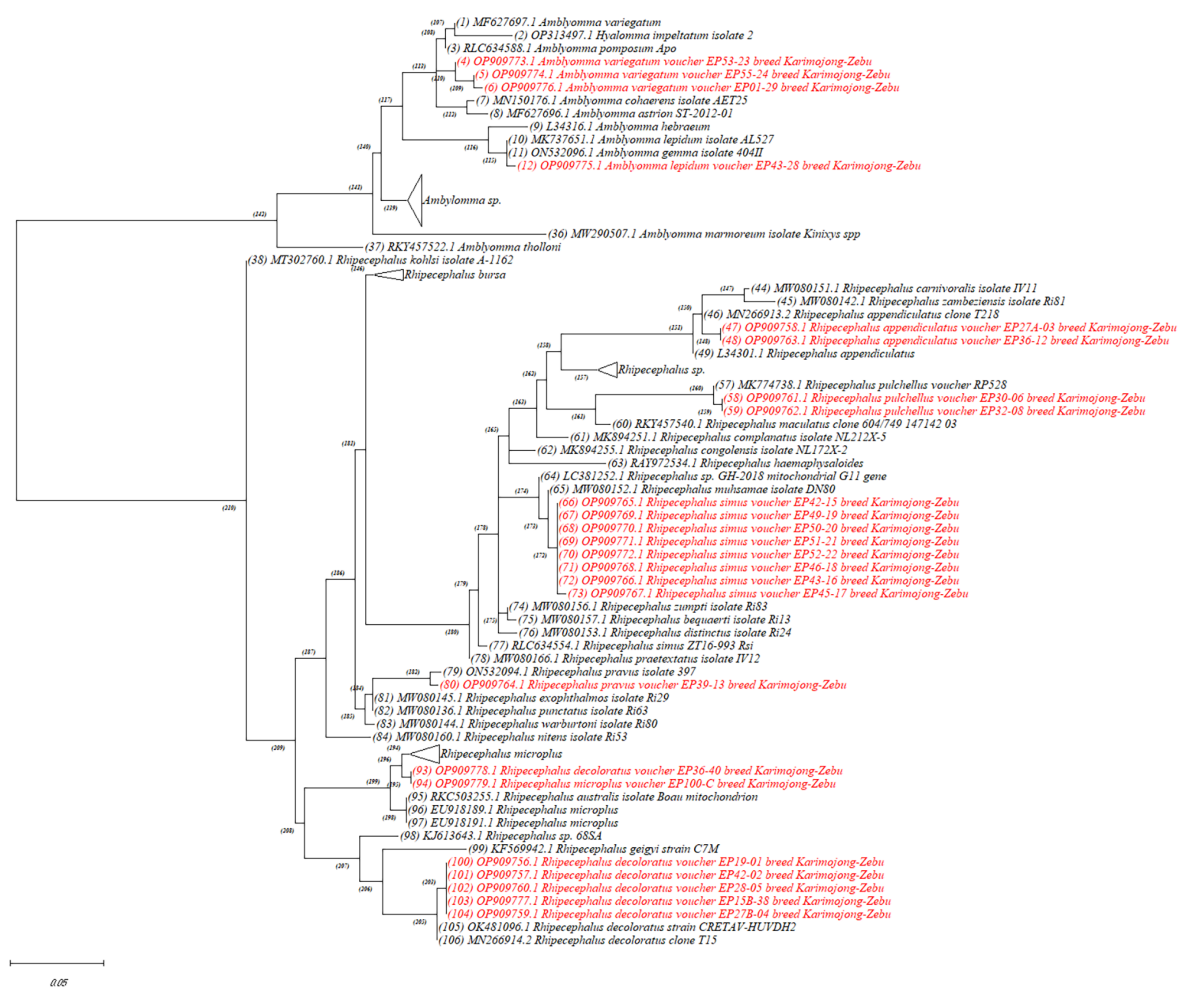
Maximum likelihood phylogenetic analysis of *16 S* rRNA sequences of ticks from Karamoja region. Red color samples refer to sequences generated from this study. Indicated are species names, voucher number and GenBank accession numbers

Twenty-four ticks selected from all the species identified morphologically were confirmed genetically with *16 S* rRNA gene sequencing. An additional file shows the details of the molecular identification (see Additional file 9). Molecular analysis revealed that all the ticks microscopically identified as *R. muhsamae* were closely related to *R. simus*. The 24 ticks’ *16 S* rRNA sequences representing the identified tick species in the present study and reference *16 S* rRNA sequences from GenBank database were used to infer phylogenetic relationships between the tick taxa as shown in Fig. 4. Sequence information for 24 ticks from this study was deposited in the GenBank and can be accessed under accession numbers: OP909756 – OP909779.

## Discussion

The tick species of the genus *Rhipicephalus* were the most abundant, followed by *Amblyomma* species. The dominance and high diversity of the genus *Rhipicephalus* and *Amblyomma* species on cattle is consistent with previous findings in Uganda [7, 8, 10, 27] and continental Africa [28, 29]. These findings show that tick infestation on cattle is mainly dominated by the species of these two genera, and amongst these species, are vectors of major diseases of economic importance in the region. This explains the high morbidity and mortality rates reportedly caused by mainly ECF and anaplasmosis in the region [11, 23].

*Rhipicephalus microplus*, the invasive tick species of Asian origin, was identified in ticks collected from the districts of Napak, Kaabong and Amudat districts. There has not been any reports of this tick specie in Karamoja region [7, 8]. Similarly, a systematic survey of ticks on livestock conducted over 50 years ago [6] from 1965 to 1966 did not find a single *R. microplus* in Uganda. This tick species was first recently reported in Serere District, Uganda by [27] where it was found that *R. microplus* had displaced the native *R. decoloratus*, a task that takes years to achieve [30]. Thereafter, it was reported in Gulu and Soroti districts [8], a proof of its high dispersal rate to new ecological zones [30]. In addition to its high dispersal and known invasiveness, *R. microplus* is an efficient vector of the highly pathogenic *Babesia bovis* [31, 32]. Therefore, there is a likelihood of the occurrence of severe losses in the livestock sector from the direct effects of the tick, and of babesiosis if no effective tick and TBDs control measures are instituted in Karamoja region.

*Amblyomma variegatum*, the African bont tick, was the second most dominant tick species collected and it was found in all the study districts. This finding is consistent with previous studies that reported the presence of *A. variegatum*, throughout the year, in the entire country spanning different vegetation and microclimates [3, 6–8, 10, 33]. *Amblyomma lepidum*, which is reported to thrive under arid conditions like those in Karamoja region [6, 34, 35], was one of the dominant tick species and found across all the study districts. This finding is consistent with the report by Balinandi et al. [8] who only found significant numbers of *A. lepidum* in Moroto district, while, Muhanguzi et al. [27] did not record a single *A. lepidum* specimen in the much wetter Serere District.

*Hyalomma truncatum*, *H. rufipes*, *A. gemma*, and *R. pulchellus* have commonly been encountered in dry / arid habitats [6, 8, 34, 35]. Although the habitat and distribution of these ticks is reported to include Uganda [24], these ticks were previously not reported in studies from the less arid parts of Uganda [10, 27, 36]. Recent surveys identified these species in the arid north east Karamoja region and the drier Kasese district, western Uganda [7, 8]. The seasonal migration of cattle in the Karamoja cluster (Turkana area of Kenya inclusive) could have introduced some of these tick species into the region, from Kenya and Ethiopia, where *A. gemma*, *A. lepidum*, *R. pulchellus*, and *H. truncatum* were previously identified on cattle [35, 37]. *Rhipicephalus pravus*, *R. geigyi*, *R. praetextatus*, *R. muhsamae* and the *Hyalomma* species were less frequently reported and distributed as previously reported [3, 6, 8]. *Hyalomma truncatum* and *H. rufipes* have only been reported in the arid Karamoja region of Uganda [7, 8]. Although their distribution is patchy and the dominance rates below 1%, their presence in the region is of great public health concern since *H. rufipes* is the most important vector of the virus causing Crimean-Congo hemorrhagic fever (CCHF) [24].

In general, the mean tick burden in the region was low to moderate; with the dry season mean tick burdens higher than those of the rainy season [Fig. 3]. This may not reflect the reality in the vegetation since the collection included only the visible ticks from cattle. Napak District had the highest mean tick burden compared to the other districts. Available evidence shows that the abundance and distribution of ticks are generally affected by the micro-climate and the availability of hosts [34, 37–39]. Much as Karamoja region is characterized as semi-arid, the districts of Kaabong and Amudat are much drier and of higher altitude compared to Napak which is at lower altitude and drains the highlands making it wetter than other Karamoja region districts sampled [40]. This could explain the high tick mean abundance. However, as much as micro-climate could provide a useful first approximation of the potential distribution of tick species, there are other factors like dispersal ability and vegetation cover that affect tick distribution [37]. The high tick counts in the dry season could be attributed to: (i) Movement to and aggregation of large numbers of cattle in the dry season grazing areas – the movement helps in tick dispersal while the large number of host supports the tick lifecycle; (ii) Presence of wild animals that also migrate in the dry seasons to areas with water and pastures which helps in tick dispersal and the persistence of tick populations; (iii) Inappropriate tick control measures instituted during animal migration [dry season] which permit the ticks to thrive on cattle; (iv) Stress factors in the dry season due to prolonged movement, limited feeds and water, and a harsh climate could combine and reduce the immunity of the cattle to resist ticks.

*Rhipicephalus microplus* and *R. praetextatus* have never been identified in Karamoja region before. This more exhaustive study has added two tick species to the known list of the tick species in the region; and more importantly that the invasive *R. microplus* tick species which is the most effective vector of highly pathogenic *Babesia bovis* [41, 42] now extended to the region. Given the level of transhumance pastoralism in the area, this tick species is likely to be extended to wider ecologies in and around Karamoja. Despite the limitation of the cross-sectional design of this study, the twofold rain and dry season surveys revealed a high diversity and abundance of multiple tick species infesting cattle all year round in the Karamoja region. This has both veterinary and public health significance, since most of the identified tick species like *H. truncatum*, *H. rufipes*, *R. appendiculatus*, *R. microplus*, *R. decoloratus* and *A. variegatum* are known vectors of many tick-borne infections globally [24], some of which are zoonotic.

## Conclusions

There is a high diversity and abundance of the tick species infesting cattle in Karamoja region all year round. The proportion of tick-infested cattle in the four districts ranged between 80.7 and 95.4% in both seasons. There was no significant variation in the proportions of tick-infested cattle in the two seasons. The tick *R. microplus* was reported for the first time in the Karamoja region signifying impending babesiosis outbreaks in the region unless effective tick control is instituted. Tick occurrence and prevalence data are useful in the design of targeted tick control strategies which are affordable and environmentally friendly. There is a need to determine the extent of spread of new tick species in Uganda and design effective control strategies considering that acaricide resistance has been reported in some parts of Uganda.

### Electronic supplementary material

Below is the link to the electronic supplementary material.

Supplementary Material 1

Supplementary Material 2

Supplementary Material 3

Supplementary Material 4

Supplementary Material 5

Supplementary Material 6

Supplementary Material 7

Supplementary Material 8

Supplementary Material 9

## Data Availability

Data supporting the conclusion of this article are included within the article. The newly generated tick sequences were submitted to the GenBank database under the accession numbers (**OP909756 – OP909779**). The datasets used and/or analyses during the preset study are available from the corresponding author upon reasonable request.

## Declarations

### Author details

^1^College of Veterinary Medicine Animal Resources and Biosecurity (COVAB), Makerere University, P.O. Box 7062, Kampala, Uganda. ^2^Medical Research Council/Uganda Virus Research Institute & London School of Hygiene and Tropical Medicine (MRC/UVRI & LSHTM), Research Unit, P.O. Box 49 Entebbe, Uganda. ‡ Current address: Center for Animal Health and Food Safety, University of Minnesota, St. Paul, MN 55108 USA,

## Abbreviations

*16S* rRNA: *16 S* ribosomal RNA gene
DNA: deoxyribonucleic acid
RNA: ribonucleic acid
GPS: geographical positioning system
TTBDs: ticks and tick-borne disease
NARO: National Agricultural Research Organization
