# Supplementary material for: Distribution and prevalence of ixodid tick species (Acari: Ixodidae) infesting cattle in Karamoja region of northeastern Uganda
Source: BMC Vet Res. 2024 Feb 7;20:50. doi: 10.1186/s12917-023-03802-1 (PMC10851484; doi:10.1186/s12917-023-03802-1)
Supplement: Supplementary file 1 — Supplementary Material 1 [file 12917_2023_3802_MOESM1_ESM.docx]

**Additional file 1: Table S1.** Cattle tick infestation rates in relation to season of collection per district of origin

| **District** | **Season** | **Non tick infested** | **Tick infested** | **% (95%CI)** | **Statistical analysis** |
| --- | --- | --- | --- | --- | --- |
| Amudat | Rainy | 35 | 149 | 80.7 (0.74-0.86) | χ2 = 0.0018, df = 1, P = 0.966 |
|  | Dry | 29 | 122 | 80.7 (73.5-86.7) |  |
| Kaabong | Rainy | 19 | 177 | 90.3 (85.2-94) | χ2 = 0.8598, df = 1, P = 0.354 |
|  | Dry | 15 | 195 | 92.8 (88.4-95.9) |  |
| Kotido | Rainy | 15 | 182 | 92.3 (87.7-95.6) | χ2 = 0.0489, df = 1, P = 0.825 |
|  | Dry | 14 | 185 | 92.9 (88.4-96.1) |  |
| Napak | Rainy | 9 | 189 | 95.4 (91.5-97.9) | χ2 = 0.0665, df = 1, P = 0.797 |
|  | Dry | 10 | 186 | 94.8 (90.8-97.5) |  |
