# Supplementary material for: Distribution and prevalence of ixodid tick species (Acari: Ixodidae) infesting cattle in Karamoja region of northeastern Uganda
Source: BMC Vet Res. 2024 Feb 7;20:50. doi: 10.1186/s12917-023-03802-1 (PMC10851484; doi:10.1186/s12917-023-03802-1)
Supplement: Supplementary file 2 — Supplementary Material 2 [file 12917_2023_3802_MOESM2_ESM.pdf]

**Additional file 2: Figure S1.** *Rhipicephalus appendiculatus*, adult male and female dorsal and ventral views.

***Rhipicephalus appendiculatus***

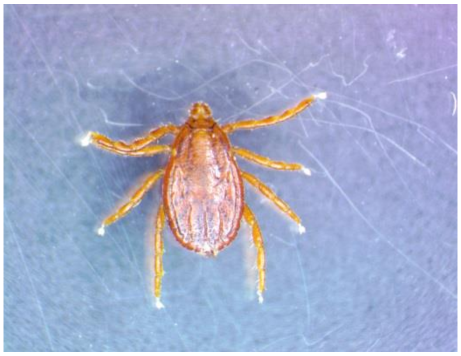

**Adult male, dorsal**

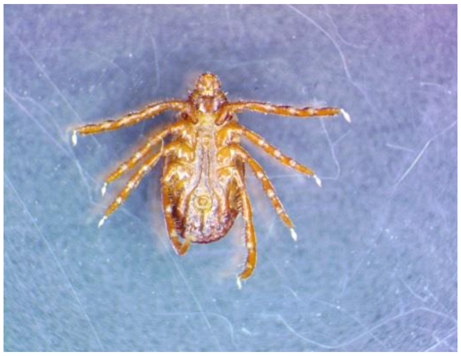

**Adult male, ventral**

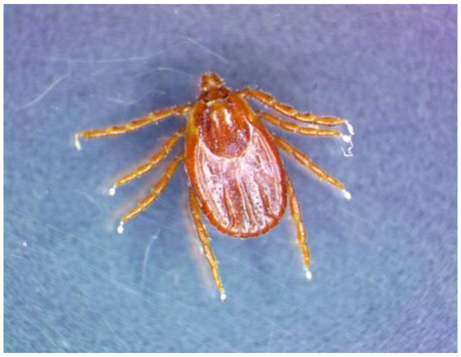

**Adult female, dorsal**

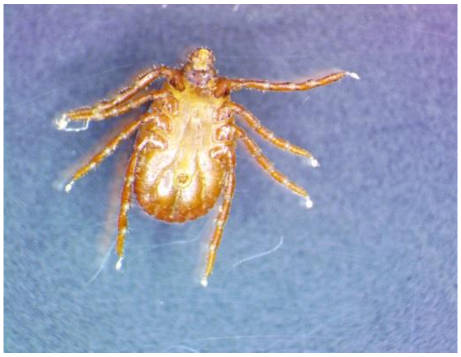

**Adult female, ventral**
