# Supplementary material for: Distribution and prevalence of ixodid tick species (Acari: Ixodidae) infesting cattle in Karamoja region of northeastern Uganda
Source: BMC Vet Res. 2024 Feb 7;20:50. doi: 10.1186/s12917-023-03802-1 (PMC10851484; doi:10.1186/s12917-023-03802-1)
Supplement: Supplementary file 4 — Supplementary Material 4 [file 12917_2023_3802_MOESM4_ESM.pdf]

Additional file 4: Figure S3. *Amblyomma gemma*, adult female and male dorsal and ventral views.

Amblyomma gemma

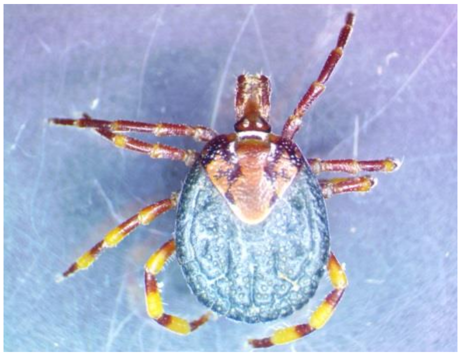

Adult female, dorsal

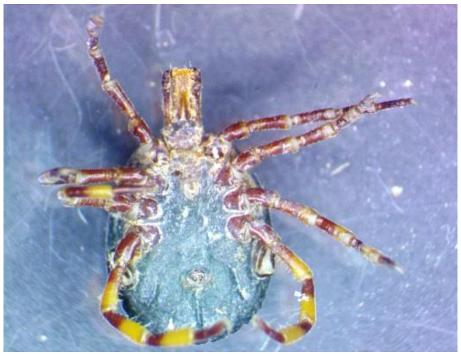

Adult female, ventral

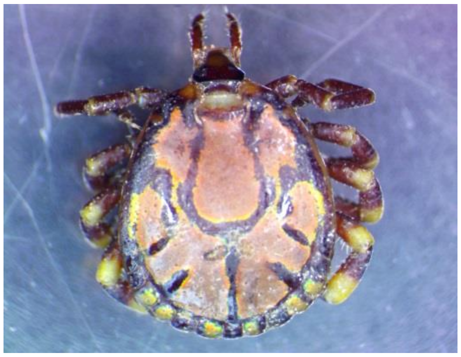

Adult male, dorsal

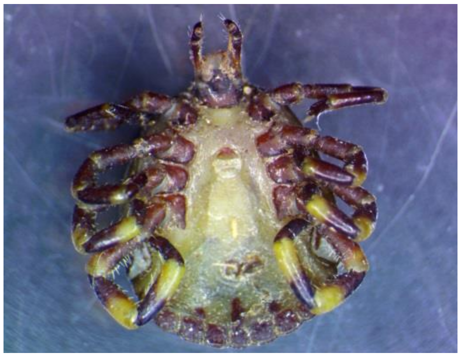

Adult male, ventral
