# Supplementary material for: Distribution and prevalence of ixodid tick species (Acari: Ixodidae) infesting cattle in Karamoja region of northeastern Uganda
Source: BMC Vet Res. 2024 Feb 7;20:50. doi: 10.1186/s12917-023-03802-1 (PMC10851484; doi:10.1186/s12917-023-03802-1)
Supplement: Supplementary file 7 — Supplementary Material 7 [file 12917_2023_3802_MOESM7_ESM.pdf]

Additional file 7: Figure S6. *Rhipicephalus simus* and *Rhipicephalus muhsamae* dorsal and ventral views.

Rhipicephalus simus

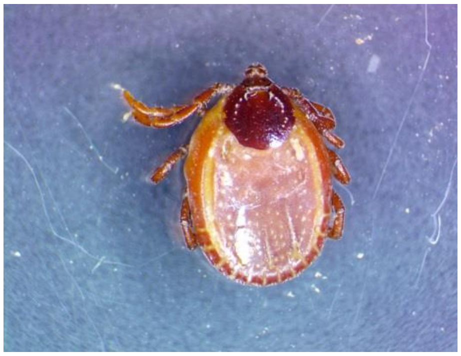

Adult male, dorsal

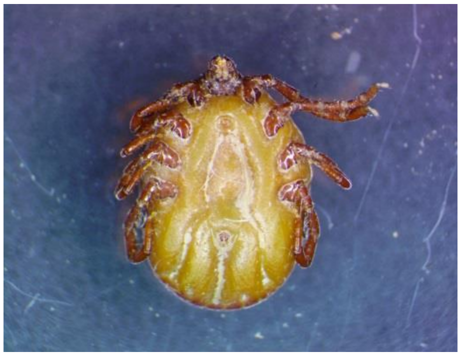

Adult male, ventral

Rhipicephalus muhsamae

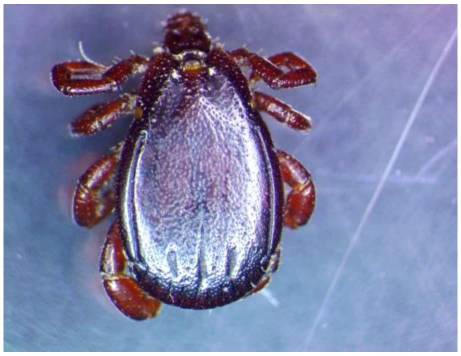

Adult male, dorsal

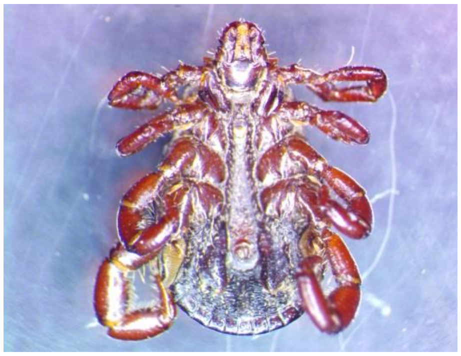

Adult male, ventral
