# Supplementary material for: Distribution and prevalence of ixodid tick species (Acari: Ixodidae) infesting cattle in Karamoja region of northeastern Uganda
Source: BMC Vet Res. 2024 Feb 7;20:50. doi: 10.1186/s12917-023-03802-1 (PMC10851484; doi:10.1186/s12917-023-03802-1)
Supplement: Supplementary file 8 — Supplementary Material 8 [file 12917_2023_3802_MOESM8_ESM.pdf]

Additional file 8: Figure S7. *Rhipicephalus evertsi* and *Rhipicephalus pravus* dorsal and ventral views.

Rhipicephalus evertsi

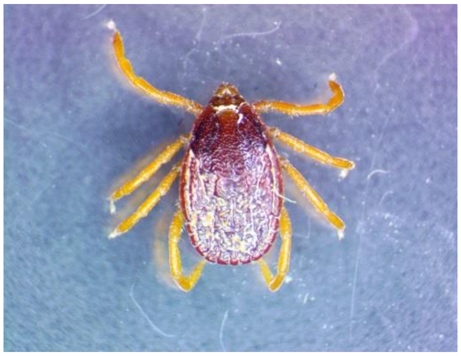

Adult male, dorsal

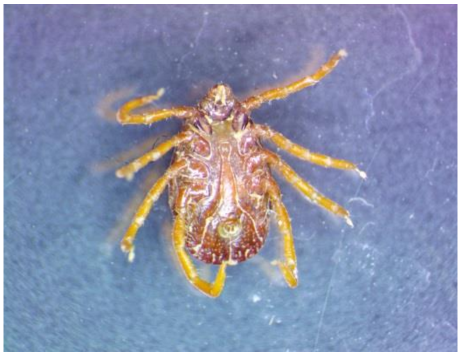

Adult male, ventral

Rhipicephalus pravus

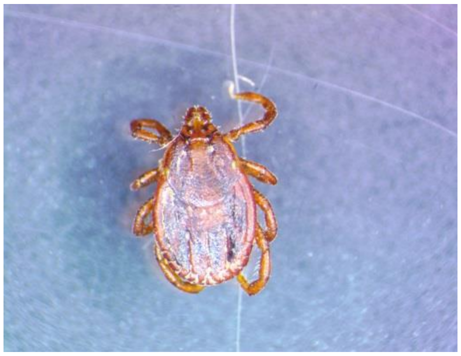

Adult male, dorsal

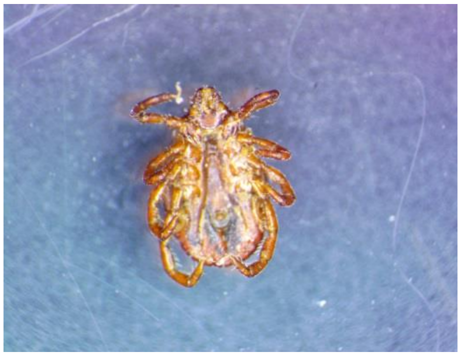

Adult male, ventral
